# Loneliness and Behavioral Changes During the COVID-19 Pandemic

**DOI:** 10.1093/geroni/igab046.2166

**Published:** 2021-12-17

**Authors:** Thomas Cudjoe, Qiwei Li, Brittany Drazich, Melissa Hladek, Laura Samuel, carl latkin, Cynthia Boyd

**Affiliations:** 1 Johns Hopkins University School of Medicine, Baltimore, Maryland, United States; 2 Johns Hopkins School of Nursing, Baltimore, Maryland, United States; 3 University of Maryland, Baltimore, Baltimore, Maryland, United States; 4 Johns Hopkins University, Baltimore, Maryland, United States; 5 Johns Hopkins Bloomberg School of Public Health, Baltimore, Maryland, United States

## Abstract

Concerns for the health impact of loneliness, a risk factor for morbidity and mortality, have risen amid the COVID-19 pandemic. However, relationships between loneliness and behavioral changes remains unclear. Utilizing data from the National Health and Aging Trends Study COVID-19 Supplement, we examine the cross-sectional relationship between loneliness and self-reported increase in each of the following behaviors during the pandemic (n=2,924): walking, vigorous activity, eating, use of alcohol and tobacco, watching television and sleeping. Adjusting for age, race, education, activity of daily living limitations, and chronic conditions, loneliness was significantly associated with a higher odds of more eating (odds ratio- OR: 1.42, confidence intervals-CI: 1.24,1.62), sleeping (OR: 1.35, CI: 1.18,1.56), and watching television (OR: 1.45, CI: 1.30,1.61). These results indicate that during stressful times like our current pandemic, loneliness may lead to morbidity and mortality through sedentary behaviors.

